# Investigating the relationships between unfavourable habitual sleep and metabolomic traits: evidence from multi-cohort multivariable regression and Mendelian randomization analyses

**DOI:** 10.1186/s12916-021-01939-0

**Published:** 2021-03-18

**Authors:** Maxime M. Bos, Neil J. Goulding, Matthew A. Lee, Amy Hofman, Mariska Bot, René Pool, Lisanne S. Vijfhuizen, Xiang Zhang, Chihua Li, Rima Mustafa, Matt J. Neville, Ruifang Li-Gao, Stella Trompet, Marian Beekman, Nienke R. Biermasz, Dorret I. Boomsma, Irene de Boer, Constantinos Christodoulides, Abbas Dehghan, Ko Willems van Dijk, Ian Ford, Mohsen Ghanbari, Bastiaan T. Heijmans, M. Arfan Ikram, J. Wouter Jukema, Dennis O. Mook-Kanamori, Fredrik Karpe, Annemarie I. Luik, L. H. Lumey, Arn M. J. M. van den Maagdenberg, Simon P. Mooijaart, Renée de Mutsert, Brenda W. J. H. Penninx, Patrick C. N. Rensen, Rebecca C. Richmond, Frits R. Rosendaal, Naveed Sattar, Robert A. Schoevers, P. Eline Slagboom, Gisela M. Terwindt, Carisha S. Thesing, Kaitlin H. Wade, Carolien A. Wijsman, Gonneke Willemsen, Aeilko H. Zwinderman, Diana van Heemst, Raymond Noordam, Deborah A. Lawlor

**Affiliations:** 1 Department of Internal Medicine, Section of Gerontology and Geriatrics, Leiden University Medical Center, PO Box 9600, 2300 RC Leiden, The Netherlands; 2 Department of Epidemiology, Erasmus MC, University Medical Center Rotterdam, Rotterdam, The Netherlands; 3 MRC Integrative Epidemiology Unit at the University of Bristol, Oakfield House, Oakfield Grove, Bristol, BS8 2BN UK; 4 Population Health Sciences, Bristol Medical School, University of Bristol, Bristol, UK; 5 Amsterdam UMC, Vrije Universiteit, Psychiatry, Amsterdam Public Health research institute, Amsterdam, The Netherlands; 6 Amsterdam Public Health Research Institute, Amsterdam, The Netherlands; 7 Department of Biological Psychology, Vrije Universiteit Amsterdam, Amsterdam, The Netherlands; 8 Department of Human Genetics, Leiden University Medical Center, Leiden, The Netherlands; 9 Department of Experimental Vascular Medicine, Amsterdam UMC, University of Amsterdam, Amsterdam, The Netherlands; 10 Human and Animal Physiology, Wageningen University, Wageningen, The Netherlands; 11 Department of Epidemiology, Mailman School of Public Health, Columbia University, New York, USA; 12 Department of Epidemiology and Biostatistics, School of Public Health, Imperial College London, London, UK; 13 NIHR Oxford Biomedical Research Centre, Oxford University Hospitals Foundation Trust, Oxford, UK; 14 Radcliffe Department of Medicine, Oxford Centre for Diabetes, Endocrinology, and Metabolism, University of Oxford, Oxford, UK; 15 Department of Clinical Epidemiology, Leiden University Medical Center, Leiden, The Netherlands; 16 Molecular Epidemiology, Department of Biomedical Data Sciences, Leiden University Medical Center, Leiden, The Netherlands; 17 Department of Internal Medicine, Division of Endocrinology, Leiden University Medical Center, Leiden, The Netherlands; 18 Department of Neurology, Leiden University Medical Center, Leiden, The Netherlands; 19 Dementia Research Institute at Imperial College London, London, W2 1PG UK; 20 MRC Centre for Environment and Health, School of Public Health, Imperial College, London, UK; 21 Einthoven Laboratory for Experimental Vascular Medicine, Leiden University Medical Center, Leiden, The Netherlands; 22 Robertson Center for Biostatistics, University of Glasgow, Glasgow, UK; 23 Department of Cardiology, Leiden University Medical Center, Leiden, The Netherlands; 24 Netherlands Heart Institute, Utrecht, The Netherlands; 25 Department of Public Health and Primary Care, Leiden University Medical Center, Leiden, The Netherlands; 26 BHF Glasgow Cardiovascular Research Centre, Faculty of Medicine, Glasgow, UK; 27 Department of Psychiatry, Groningen, University of Groningen, University Medical Center Groningen, Groningen, The Netherlands; 28 Max Planck Institute for Biology of Ageing, Cologne, Germany; 29 Department of Clinical Epidemiology, Biostatistics, and Bioinformatics, Amsterdam UMC, University of Amsterdam, Amsterdam, The Netherlands; 30 NIHR Bristol Biomedical Research Centre, Bristol, UK

**Keywords:** Mendelian randomization, Metabolomics, Sleep, Epidemiology

## Abstract

**Background:**

Sleep traits are associated with cardiometabolic disease risk, with evidence from Mendelian randomization (MR) suggesting that insomnia symptoms and shorter sleep duration increase coronary artery disease risk. We combined adjusted multivariable regression (AMV) and MR analyses of phenotypes of unfavourable sleep on 113 metabolomic traits to investigate possible biochemical mechanisms linking sleep to cardiovascular disease.

**Methods:**

We used AMV (*N* = 17,368) combined with two-sample MR (*N* = 38,618) to examine effects of self-reported insomnia symptoms, total habitual sleep duration, and chronotype on 113 metabolomic traits. The AMV analyses were conducted on data from 10 cohorts of mostly Europeans, adjusted for age, sex, and body mass index. For the MR analyses, we used summary results from published European-ancestry genome-wide association studies of self-reported sleep traits and of nuclear magnetic resonance (NMR) serum metabolites. We used the inverse-variance weighted (IVW) method and complemented this with sensitivity analyses to assess MR assumptions.

**Results:**

We found consistent evidence from AMV and MR analyses for associations of usual vs. sometimes/rare/never insomnia symptoms with lower citrate (− 0.08 standard deviation (SD)[95% confidence interval (CI) − 0.12, − 0.03] in AMV and − 0.03SD [− 0.07, − 0.003] in MR), higher glycoprotein acetyls (0.08SD [95% CI 0.03, 0.12] in AMV and 0.06SD [0.03, 0.10) in MR]), lower total very large HDL particles (− 0.04SD [− 0.08, 0.00] in AMV and − 0.05SD [− 0.09, − 0.02] in MR), and lower phospholipids in very large HDL particles (− 0.04SD [− 0.08, 0.002] in AMV and − 0.05SD [− 0.08, − 0.02] in MR). Longer total sleep duration associated with higher creatinine concentrations using both methods (0.02SD per 1 h [0.01, 0.03] in AMV and 0.15SD [0.02, 0.29] in MR) and with isoleucine in MR analyses (0.22SD [0.08, 0.35]). No consistent evidence was observed for effects of chronotype on metabolomic measures.

**Conclusions:**

Whilst our results suggested that unfavourable sleep traits may not cause widespread metabolic disruption, some notable effects were observed. The evidence for possible effects of insomnia symptoms on glycoprotein acetyls and citrate and longer total sleep duration on creatinine and isoleucine might explain some of the effects, found in MR analyses of these sleep traits on coronary heart disease, which warrant further investigation.

**Supplementary Information:**

The online version contains supplementary material available at 10.1186/s12916-021-01939-0.

## Background

Several systematic reviews and large biobank studies have reported associations of self-reported insomnia symptoms, short and long sleep duration, and chronotype (i.e. having an evening rather than morning preference) with increased risk of cardiovascular disease, type 2 diabetes, and risk factors for these [1–9]. The mechanisms underlying these associations are unclear, and it is plausible that specific sleep traits may contribute to the misalignment of various behavioural and internal physiological processes, including aspects of metabolism that causes adverse cardiometabolic health.

There is some evidence of poor sleep quality, shorter sleep duration, and having an evening chronotype being associated with higher triglyceride, total cholesterol and low-density lipoprotein cholesterol (LDL-C) levels, and lower high-density lipoprotein cholesterol (HDL-C) concentrations [10–12]. However, the extent to which these associations are explained by confounding factors, such as body mass index [11], is unclear. Beyond conventional multivariable-adjusted regression analyses, we have previously demonstrated that sleep duration modifies the associations of genetic variation with triglycerides, LDL-C and HDL-C in a large sleep-gene interaction analysis, suggesting that possible different biological mechanisms underlie the associations of short and long sleep duration with these lipid traits [13]. However, these genetic interaction analyses do not assess causality and, like previous multivariable-adjusted regression analyses, have focused on a limited number of lipid traits.

Mendelian randomization (MR) uses genetic variants that are robustly associated with an exposure as an instrumental variable to obtain unconfounded effects of that exposure on an outcome of interest [14–16]. Recent MR analyses have suggested a causal effect of insomnia symptoms on coronary heart disease [17] and of short (< 6 h) sleep duration on myocardial infarction risk [18].

The aim of this study was to determine the possible causal effect of sleep traits on metabolomic traits. We compared findings from adjusted multivariable regression (AMV) and MR analysis, to determine the relationships between self-reported insomnia symptoms (usually vs. sometimes/rare/never), total habitual sleep duration (per 1 h longer), and chronotype (evening vs. morning preference) and 113 nuclear magnetic resonance (NMR) metabolomic traits. Cross-sectional AMV was performed with adjustment for age, sex, and BMI in 17,370 individuals from 10 cohorts of mostly Europeans. Two-sample MR used summary results from genome-wide association studies (GWAS) of different sleep traits in 1,331,010 (insomnia) [19], 446,118 (sleep duration) [20], and 651,295 (chronotype) [21] European adults and summary results from four GWAS of 113 circulating metabolomic measures from NMR in 38,618 European adults. In secondary analyses, we explored effects of short (< 7 vs. 7- < 9 h) and long (≥ 9 vs. 7- < 9 h) sleep duration on the metabolomic traits. We highlight results that were consistent across both methods, as the different key sources of bias of the two methods (e.g. residual confounding in AMV and unbalanced horizontal pleiotropy in MR, respectively) mean that, where there is consistency, this is more likely to reflect a causal effect [22].

## Methods

### Studies used for AMV

Cross-sectional AMV analyses were performed using data from 10 cohorts: the Active and Healthy Ageing (AGO) study [23], the Dutch Hunger Winter Families Study (DWFS) [24], the Healthy Life in an Urban Setting (HELIUS) Study [25], the Leiden University Migraine Neuro-Analysis (LUMINA) [26], Netherlands Study of Depression and Anxiety (NESDA) [27], the Netherlands Twin Register (NTR) [28], the Netherlands Epidemiology of Obesity (NEO) Study [29], and the Rotterdam Study cohorts 1, 2, and 3 (RS1, RS2 and RS3) [30]. Study characteristics of each study are given in Additional file 1: Table S1. Each participating study obtained written informed consent from all participants and received approval from the appropriate local institutional review boards. Before the analyses, we excluded all participants with diabetes (defined as self-report/hospital record, fasting plasma glucose > 7 mmol/L and/or use of hypoglycaemic medication) given the known disturbances on many metabolomic traits. In more detail:

#### Active and Healthy Ageing (AGO) Study

The “Actief en Gezond Oud (AGO)” study is a randomized controlled trial of the effect of a 3-month Web-based intervention program with the intention to improve physical activity in inactive older adults. A more detailed description of the study setting and selection of study participants is described in more detail elsewhere [23]. In short, individuals were eligible for study inclusion when they were between 60 and 70 years of age, had no history of diabetes or use of glucose-lowering mediation, had no disabilities impending increase in physical activity, and were in the possession of a personal computer with access to the internet. All eligible individuals were screened for the presence of an inactive lifestyle using the general practice physical activity questionnaire (GPPAQ). Eligible individuals with an active lifestyle were not included in the study. After study inclusion, participants were randomized into an intervention and control (waitlist) group. For the present study, we only included the baseline sample prior to randomization during which information on sleep was collected using the PSQI questionnaire and fasting blood was taken. In the final sample for the present study, we included 221 participants.

#### Healthy Life in an Urban Setting (HELIUS)

The HELIUS study is a prospective cohort study among six large ethnic groups living in Amsterdam, the Netherlands. Between 2011 and 2015, a total 24,789 participants (aged 18–70 years) were included at baseline [31, 32]. Similar-sized samples of individuals of Dutch, African Surinamese, South-Asian Surinamese, Ghanaian, Turkish, and Moroccan origin were included using stratified random sampling from the Amsterdam municipal records. Response rate was about 28% of those invited and 50% of those with whom some form of contact was established.

Participants filled in an extensive questionnaire and underwent a physical examination that included the collection of biological samples (biobank). Participants were asked to provide information on the average number of hours they usually sleep at night. Sleep duration was assessed using the item “How many hours do you sleep on average per night?” Sleep duration was categorized according to the standard recommendations of the National Sleep Foundation. For adults, 7–9 h per night is recommended. Short sleep is defined as having less than 7 h of sleep per night and long sleep as having 9 or more hours of sleep per night. Venous blood samples were obtained after overnight fasting (minimum 4 h), processed within 4 h and then stored at − 80 °C. Samples were freeze-thawed no more than 1 time prior to shipment. For the present study, 500 participants with African-Surinamese or Ghanaian ethnicity living in the Netherlands with pre-diabetes were included.

#### The Dutch Hunger Winter Families Study (DHWF)

DHWF consists of 2417 singleton births with detailed birth records, born between 1 February 1945 and 31 March 1946 to mothers who were exposed to the Dutch famine of 1944–1945 during or immediately preceding pregnancy and an additional 890 births that occurred between 1943 and 1947 and who were selected on the basis that their mothers were not exposed to famine during this pregnancy. For 70% of the individuals, an address could be obtained, and they were invited to participate together with a same-sex sibling not exposed to the famine. In total, 1075 (33% of original identified births) interviews and 971 (29%) clinical examinations were performed between 2003 and 2005. Fasting (minimum 9 h) venous blood samples were obtained, and then stored at − 80 °C. Samples were freeze-thawed no more than one times prior to shipment. Sleep habits were ascertained as per NHANES I questionnaire during hospital interview at the Leiden University Medical Center [24]. A total of 963 participants with data on sleep traits and nuclear magnetic resonance (NMR) metabolites were included in analyses presented in this paper.

#### The Leiden University Migraine Neuro-Analysis (LUMINA)

Participants of the Leiden University Migraine Neuro-Analysis (LUMINA) study were recruited through a dedicated, nationwide website inviting migraine patients and non-migraine controls to participate in migraine research. Additional participants were recruited from patients attending the Leiden University Medical Center (LUMC) dedicated headache clinic. Blood venous samples were drawn and after centrifugation at room temperature plasma was aliquoted and stored at − 80 °C. Samples were freeze-thawed no more than once prior to NMR analyses. Sleep was assessed using the Pittsburgh Sleep Quality Index (PSQI), the Munich ChronoType questionnaire (MCTQ) and the Insomnia Severity Index (ISI). In total, 248 participants with sleep and NMR data were included in the analyses presented here.

#### The Netherlands Study of Depression and Anxiety (NESDA)

NESDA is an observational longitudinal cohort study on the long-term course and consequences of depressive and anxiety disorders [27]. In total, 2981 participants aged 18 to 65 years were recruited between 2004 and 2007 through different settings: community, primary care, and specialized mental health clinics in order to obtain a representative sample of persons with and without depressive and anxiety disorders. Plasma samples were obtained at baseline, stored in the EDTA detergent, stored at − 80 °C until further analyses, and shipped in two batches (April and December 2014, further referred to as batch 1 and batch 2, respectively) to the NMR metabolomics lab for assessment. Insomnia symptoms were based on the 4-item Women’s Health Insomnia Rating Scale. This questionnaire addresses trouble falling asleep, waking up during the night, early morning awakenings, trouble getting back to sleep after waking up, and sleep quality. Sleep duration was based on one self-report question. At the 2-year follow-up assessment, chronotype was based on the Munich Chronotype Questionnaire.

In total, 2483 respondents with sleep information and NMR metabolomics data were included.

#### The Netherlands Twin Register (NTR)

The NTR (http://www.tweelingenregister.org/) has collected (longitudinal) data on young and adult twins and their families [28, 33]. A 2015 estimate suggests that the NTR includes ~ 25% (~ 2,000,000 individual participants, including family members of twins/multiples). An initial NTR biobank project (BB1) obtained blood samples from 9530 participants, from 3477 families, via home visits between January 2004 and July 2008. A second project (BB2) collected blood samples from 517 participants between January 2011 and December 2011. This sample included 210 MZ twin pairs and 64 twin-spouse pairs [4]. Visits were scheduled between 7:00 am and 10:00 am to collect fasted (overnight) venous samples (fertile women were bled on days 2–4 of the menstrual cycle or in their pill-free week). Samples were stored at − 80 °C. Sleep variables were assessed using the Dutch Groningen Sleep Questionnaire [34]. In total, 3398 participants with sleep and NMR data were included in the analyses presented here.

#### The Netherlands Epidemiology of Obesity (NEO) Study

The NEO study is a prospective population-based cohort study. In this paper, we used cross-sectional data obtained at the baseline assessment. The NEO study started in 2008 and included 6671 individuals aged 45–65 years, with an oversampling of individuals with a BMI of 27 or higher. The study design and population are described in more detail elsewhere [29]. Men and women living in the greater area of Leiden (in the West of the Netherlands) were invited to participate if they were aged between 45 and 65 years and had a self-reported body mass index (BMI) of 27 kg/m^2^ or higher. In addition, all inhabitants aged between 45 and 65 years from one municipality (Leiderdorp) were invited to participate irrespective of their BMI, allowing for a reference group with a normal BMI distribution. Data on sleep was collected using the standardized PSQI questionnaire and fasting blood was collected on the baseline visit to the study centre. A total of 5094 participants had complete data and contributed to the present analysis.

#### The Rotterdam Study

From 1989, all inhabitants aged 55 and older from a well-defined suburb in the city of Rotterdam, the Netherlands, were invited to participate in the Rotterdam Study. The initial cohort comprised 7983 (78% of those invited) participants (RS-I) and was extended in 2000 (RS-II: 3011 participants (67%)) and 2006 (RS-III: 3932 participants (65%), aged 45 years and older). In total, the Rotterdam Study comprises 14,926 participants aged 45 years or over. The Rotterdam Study has been registered at the Netherlands National Trial Register (NTR; www.trialregister.nl) and the WHO International Clinical Trials Registry Platform (ICTRP; www.who.int/ictrp/network/primary/en/) under shared catalogue number NTR6831.

Between 2002 and 2014, overnight fasted venous blood samples were obtained and analysed using NMR technique from 5381 participants across all three cohorts. Samples were aliquoted and then stored at − 80 °C. Samples were not freeze-thawed prior to shipment to Brainshake Ltd./Nightingale Health for NMR analyses. Sleep traits were measured during a home interview using the Pittsburgh Sleep Quality Index (PSQI). The assessment of chronotype was based on a single question from a sleep diary. A total of 4730 participants from across the three cohorts with data on sleep traits and NMR were included in this study.

### Studies used for MR analyses

We performed two-sample MR analyses using publicly available summary-level data [14] from the following GWAS:

### Sleep trait GWAS

We selected genome-wide significant (*p* value< 5e−8) variants as instrumental variables from the following GWAS. All associations had been adjusted for age, sex, a maximum of 10 principal components, and, additionally, in UK Biobank for genotype platform:

- Insomnia: A GWAS that pooled data from two large biobanks (UK Biobank and 23andMe) and included 1,331,010 unrelated European-ancestry adults. This GWAS identified 248 variants (Additional file 1: Table S2; total F-statistic = 6918) for experience of insomnia symptoms (usually vs. sometimes/rare/never) [19].
- Sleep duration: A GWAS undertaken in UK Biobank of 446,118 unrelated European-ancestry adults [20]. This GWAS identified 78 variants for total sleep duration (mean 7.2 h; SD1.1 h; Additional file 1: Table S3; total F-statistic = 2567). In addition, this GWAS identified 27 variants for short sleep duration (< 7 h vs. 7 to < 9 h; *N* = 106,192 cases; Additional file 1: Table S4; total F-statistic = 646) and 8 for long sleep duration (≥ 9 h vs. 7 to < 9 h; *N* = 34,184 cases; Additional file 1: Table S5; total F-statistic = 208).
- Chronotype: A GWAS that pooled data from two large biobanks (UK Biobank and 23andMe) and included 697,828 unrelated European-ancestry adults (651,295 of whom were in the combined (both biobanks) GWAS of morning versus evening preference that we have used in this two-sample MR study. This GWAS identified 351 variants for chronotype [21] (Additional file 1: Table S6; total F-statistic = 13,967). Because previous observational studies have found increased risk of cardiometabolic diseases and risk factors in those with an evening preference, we transformed the GWAS results to reflect alleles associated with evening preference.

### NMR Metabolite GWAS

- MAGNETIC consortium (*N* = 24,925) [35] with summary-level GWAS data downloaded from http://www.computationalmedicine.fi/data#NMR_GWAS
- In addition, to increase statistical power in the MR analyses, we generated new summary-level GWAS data from three cohorts using similar analyses procedures to the MAGNETIC consortium: Oxford Biobank (*N* = 6616) [36], NEO (*N* = 4734) [29], and Pravastatin in Elderly Individuals at Risk of Vascular Disease (PROSPER) (*N* = 2343; placebo arm only) [37].

All GWASs were undertaken in participants of European ancestry, and there was no overlap between the cohorts included in the sleep trait GWAS and those included in the NMR GWAS. In more detail:

#### MAGNETIC consortium

We used publicly available summary statistics from the MAGNETIC NMR GWAS dataset (downloaded from: http://www.computationalmedicine.fi/data#NMR_GWAS), which comprises the additive (per-allele) beta coefficients with accompanying standard errors of the associations between genome-wide single nucleotide polymorphisms (SNPs) and 123 metabolic measures [35]. This GWAS meta-analysed data from 24,925 European ancestry participants of 14 cohorts. The 123 metabolic measures in the studies included in the MAGNETIC NMR GWAS were quantified by an earlier version of the same high-throughput proton NMR metabolomics platform as that used in the multivariable regression studies meta-analysis (https://nightingalehealth.com/research/blood-biomarker-analysis).

In addition to the publicly available data from the MAGNETIC consortia, we were also able to obtain summary GWAS data for the same NMR platform metabolites from 3 additional cohorts. GWAS analyses in these additional cohorts were run by co-authors for this paper and these results have not yet been published/made public. In each GWAS, only those of European ancestry were included, metabolites were log-transformed and results were the per-allele difference in mean metabolite in standard deviation (SD) units of the logged variables. Additive linear regression analyses were performed adjusted for age, sex and the first 10 principal components to correct for population stratification. Descriptions of these three studies are provided below.

#### NEO Study

The general description of the NEO study is provided above. Genotyping was performed in participants form European ancestry, using the Illumina HumanCoreExome-24 BeadChip (Illumina Inc., San Diego, California, USA). Related individuals as well as individuals of a non-European ancestry were excluded for genotyping [38]. Subsequently, genotypes were imputed to the 1000 Genome Project reference panel (v3 2011). 4734 NEO participants were included in the GWAS that provided data for this study.

#### Oxford Biobank (OBB)

OBB is a population-based cohort study of randomly selected healthy men and women living in Oxfordshire, UK. The study includes 7185 individuals aged 30 to 50 years old. The exclusion criteria for the OBB were history of myocardial infarction, diabetes mellitus type 1 or 2, heart failure, untreated malignancy, other ongoing systemic diseases, or ongoing pregnancy. Study recruitment criteria and population characteristics are described in detail elsewhere [36]. OBB was approved by the Oxfordshire Clinical Research Ethics Committee and all participants provided informed consent. SNP array data have been generated using the Illumina Infinium Human Exome Beadchip 12v1 array platform for the first consecutive 5900 DNAs, and Affymetrix UK Biobank Axiom Array chip on the first consecutive 7500 participants. A total of 6616 participants were included in the GWAS that provided data for this study.

#### PROSPER

PROSPER is a prospective multicentre randomized placebo-controlled trial that was established to determine the effect of pravastatin (a statin) on the risk of major vascular events in elderly adults. Between December 1997 and May 1999, potential participants were screened and enrolled in Scotland (Glasgow), Ireland (Cork), and the Netherlands (Leiden). Men and women aged 70–82 years were recruited if they had pre-existing vascular disease or increased risk of such disease because of smoking, hypertension, or diabetes [37, 39]. A total of 23,770 individuals were assessed for eligibility. A total number of 5804 (24.4% of the invited eligible participants) adults were randomly assigned to pravastatin or placebo. Participants were followed for an average 3.5 years. Genotyping was performed using the Illumina Beadchip 660 K. Outlying individuals were excluded on the basis of relatedness, non-European ancestry, and sex discrepancy. Genotyped data was subsequently imputed to the HRC reference panel. 2343 PROSPER participants were included in the GWAS that provided data for this study. Analyses were adjusted for age, sex, and the first 10 principal components to correct for population stratification.

### Sleep traits

In both AMV and MR analyses, sleep traits were self-reported and analysed in the same units/categories. Contributing cohorts either collected some individual question on habitual sleep duration (e.g. HELIUS) or collected more aspects of sleep using the PSQI questionnaire. Insomnia symptoms were assessed with a question similar to “Do you have trouble falling asleep at night or do you wake up in the middle of the night?” with the following answers possible: “never/rarely”, “sometimes”, “usually”, or “prefer not to answer”. In the AMV and GWAS analyses, participants who answered “usually” were defined as having insomnia symptoms and were compared to those answering “never/rarely” or “sometimes”. Habitual sleep duration was assessed using a question similar to “On an average day, how many hours of sleep do you get?”. For our main analyses, we examined effects of total self-reported sleep duration (per 1 h longer) on metabolomic measures. In secondary analyses, we explored associations of short (< 7 vs. 7- < 9) and long (≥ 9 vs. 7- < 9 h) habitual sleep. These latter two analyses were considered exploratory because of lower statistical power and possible weak instrument bias in the MR analyses. For chronotype, a question similar to “Are you naturally a night person or a morning person?” with the possible responses “Night owl/night person”, “Early bird/morning person”, “Neither/not sure” was used in most studies. A variation on the question in UK Biobank included more responses: “Definitely a morning person”, “More a morning than evening person”, “More an evening than a morning person”, “Definitely an evening person”, “Do not know”. Participants were classified as having a ‘morning preference’ (“Early bird/morning person”, “Definitely a morning person” or “More a morning than evening person”), the reference group, or an ‘evening preference’ (“Night owl/night person”, “More an evening than a morning person” or “Definitely an evening person”). For all traits those responding “do not know”, “unsure” or “prefer not to answer” were excluded.

### NMR-based metabolomic profiling

In both the metabolite GWAS and studies included in the AMV meta-analysis, metabolites were quantified using a high-throughput proton (^1^H) NMR metabolomics platform [40] (https://nightingalehealth.com/) to quantify a maximum of 148 (excluding ratios) lipid and lipoprotein and metabolite concentrations in fasting serum or plasma samples. The quantitative NMR measures include numerous lipid species and fatty acids, as well as some amino acids, markers of glucose homeostasis, fluid balance, and an inflammatory marker. This platform has been used widely in population-based studies of cardiometabolic diseases and has been described in detail elsewhere [40–42]. There were 113 metabolomic trait measurements that were available for both AMV and MR analyses.

### Statistical analyses

In both AMV and MR analyses, we estimated the same effect: the difference in mean NMR metabolites (SD units of the natural log-transformed metabolomic traits; as dependent variables) comparing (i) usually experiencing insomnia symptoms to sometimes, rarely or never, (ii) per 1 h longer habitual sleep duration, and (iii) an evening to a morning preference. All analyses were performed in R (v3.6.1) [43].

#### Multivariable-adjusted regression meta-analysis

Cross-sectional AMV was performed by each of the individual cohorts according to a pre-specified analysis plan and standardized analysis script. Results were collected centrally for quality control subsequent fixed-effect meta-analyses using the R “rmeta” package, using similar procedures as described previously [44]. Additionally, we performed random-effect meta-analyses using the same software to incorporate possible between-cohort heterogeneity. AMV analyses adjusted for age, sex, and BMI.

#### Mendelian randomization analyses

We excluded all palindromic single nucleotide polymorphisms (SNPs) and those in linkage disequilibrium at *R*^2^ > 0.001 (based on the 1000genomes (phase 1) panel). After these exclusions, we searched for all remaining independent sleep-associated variants (149 for insomnia, 57 for total sleep duration [and an additional 25 and 71 variants for short and long habitual sleep duration, respectively] and 208 for chronotype) in the GWAS of NMR metabolomic measures, and the directions of the summary data were harmonized (i.e. making sure that each effect estimate was coded in the same direction with respect to the effect allele as SNP associations from the summary sleep trait data) with those of the sleep trait summary data.

The MRCIEU/TwoSampleMR package was used for harmonization of the exposure and outcome SNPs and to perform the MR analyses [16]. For our main analyses, we used the multiplicative random effects inverse variance-weighted (IVW) approach [45]. This method generates a causal estimate of the sleep traits on metabolomic traits by regressing the SNP-sleep trait association on the SNP-metabolomic measure association, weighted by the inverse of the SNP-metabolomic measure association, and constraining the intercept of this regression to zero. Standard errors are corrected to take into account any between SNP heterogeneity and assume that there is no directional horizontal pleiotropy. To explore this assumption further, we performed sensitivity analyses using MR-Egger [46] and weighted-median estimator [47] methods. MR-Egger is similar to the IVW method but does not force the regression line (i.e. of the SNP-sleep trait association on the SNP-metabolomic measure association) through an intercept of zero. It is statistically less efficient (providing wider confidence intervals) but provides a causal estimate (i.e. the regression slope) that is corrected for directional horizontal pleiotropy, and a non-zero intercept is an indication of the existence of directional pleiotropy. The weighted-median estimator is valid if more than 50% of the weight of the genetic instrument is from valid variants (i.e. if one single SNP or several SNPs jointly contributing 50% or more of the weight in the MR analysis exhibit directional horizontal pleiotropy the calculated effect estimate may be biased). For each of the dataset, we assessed between-SNP heterogeneity using the Q-statistics test.

We performed MR analyses for all sleep traits with each of the 4 metabolomic GWAS data sources (MAGNETIC, Oxford Biobank, NEO, and PROSPER), and the results were subsequently meta-analysed using fixed-effect meta-analyses as implemented in the R package rmeta.

### Comparing multivariable regression and MR analysis results

Circos plots were used to summarize and visually compare the AMV and the IVW MR results. Circos plots were created using EpiViz (version 0.1.0, https://github.com/mattlee821/EpiViz/), a Shiny web application and R package built using R (version 3.6.2), and Shiny (version 1.4.0). Shiny is an R package that enables development and deployment of web applications written in the R programming language. EpiViz adapts and builds on the Circlize [48] and ComplexHeatmap [49] R packages to create Circos plots compatible with association analysis data.

We also generated scatter plots of the AMV vs. MR results for each metabolite and compared the linear fit across all metabolites to a slope of perfect concordance and used *R*^2^ as a measure of goodness of fit (agreement) between the two methods across all 113 metabolomic traits.

Having compared results for the AMV and IVW MR methods across all metabolites, we then selected all sleep trait-metabolite associations that reached a pre-defined *p* value threshold in AMV or IVW MR. We then compared results across AMV, IVW MR, MR-Egger, and weighted median MR for those selected associations. Whilst we focus on results reaching a pre-defined *p* value threshold in either AMV or IVW MR in the main paper and our conclusion, a full set of all results (AMV, unadjusted MV, IVW MR, and all MR sensitivity analyses) are presented in Additional file 1: Tables S7 to S16. We applied the same Bonferroni multiple testing corrected *p* value threshold separately to the AMV and MR analyses. The threshold was determined taking into account the correlation structure of the metabolomic measures by using information from previous studies that have identified 17 principal components, which explain 95% of the metabolomic traits data variance [50]. Therefore, the two-sided threshold of *P <* 0.05 adjusted for multiple testing becomes *P <* 0.0029 (0.05/17). For any association that passed this threshold with either AMV or IVW MR, we considered the result from the second method to be consistent if the point estimate had a similar direction of effect and the *p* value for the second association was *<* 0.05. This was justified on the basis that once one method passed the Bonferroni threshold, we were treating that result as a hypothesized effect and seeking replication and triangulation in the second method.

## Results

Full results of all AMV and MR analysis, including MR sensitivity analysis results, are presented in Additional file 1: Tables S7 to S16.

### Insomnia symptoms

**Fig. 1:**
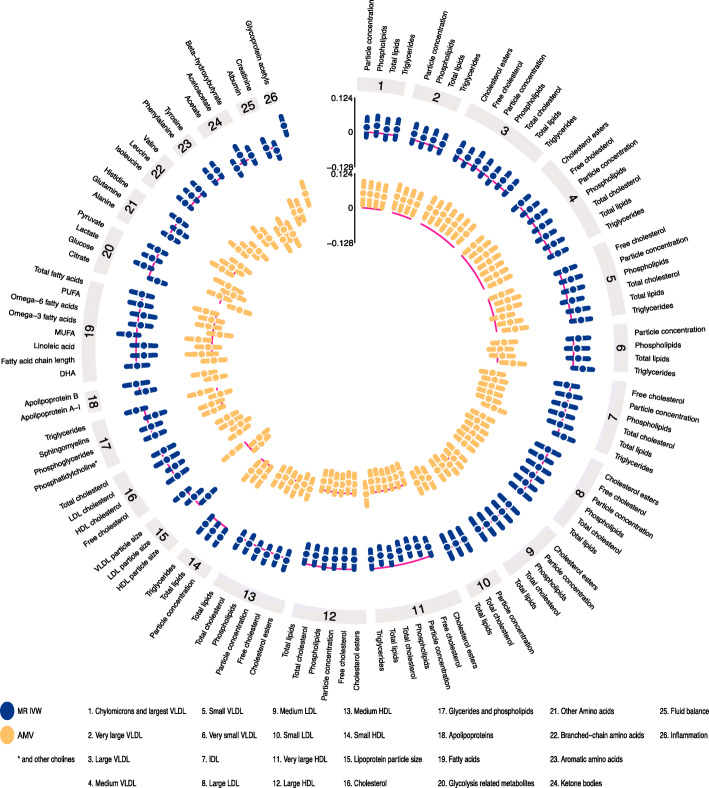
IVW Mendelian randomization estimates and age-, sex-, and BMI-adjusted multivariable regression estimates for the associations between insomnia symptoms and 113 NMR-derived metabolomic measures. Results are expressed as the difference in mean metabolite concentrations (in standard deviation units) between those reporting usually versus sometimes/rarely/never experiencing insomnia symptoms. Abbreviations: AMV, adjusted (age, sex, BMI) multivariable regression; BMI, body mass index; IDL, intermediate density lipoprotein; IVW MR, Inverse variance weighted Mendelian randomization; LDL, low-density lipoprotein; NMR, nuclear magnetic resonance; VLDL, very large density lipoprotein

**Fig. 2:**
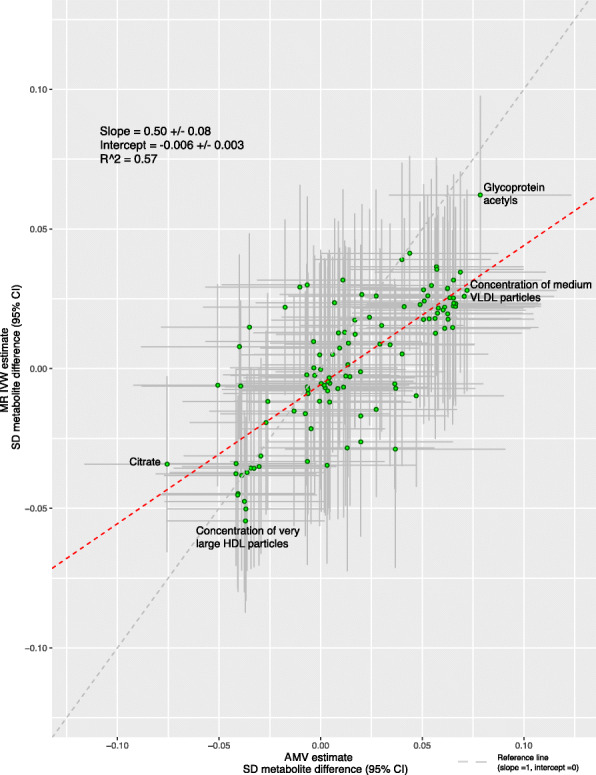
Comparison of the point estimates of the IVW Mendelian randomization and age-, sex-, and BMI-adjusted multivariable regression analyses for the associations between insomnia symptoms and 113 NMR-derived metabolomic measures. Each green dot in the scatter plot represents a metabolic trait and the positions of the dots are determined by the differences in mean metabolite concentrations (in standard deviation units) between those reporting usually versus sometimes/rarely/never experiencing insomnia symptoms. These are estimated by Inverse variance weighted (IVW) Mendelian randomization (vertical axes) and age, sex, and BMI adjusted multivariable regression (horizontal axes). The vertical grey lines for each dot indicate the 95% confidence intervals (CI) for the Mendelian randomization estimates and the horizontal grey lines for each dot indicate the 95% CI for the adjusted multivariable regression estimates. A linear fit (red dashed line) summarizes the similarity between the two estimates. A slope of 1 with an intercept of 0 (dashed grey line), with all green dots sitting on that line (*R*^2^ = 1), would indicate identical magnitude and direction between the two methods. *R*^2^ indicates goodness of linear fit and is a measure of the consistency between the two estimates. Abbreviations: AMV, adjusted (age, sex, BMI) multivariable regression; BMI, body mass index; CI, confidence interval; DHA, 22:6, docosahexaenoic acid; IVW MR, inverse variance weighted Mendelian randomization, SD, standard deviation

Visually inspecting the circos plot shows there was directional consistency between the AMV and IVW MR results for most of the metabolomic traits (Fig. 1). With both methods, insomnia symptoms were associated with higher concentrations of small and medium very large density lipoprotein (VLDL) particles, small HDL particles and glycoprotein acetyls, and with lower concentrations of large HDL particles. Across all 113 metabolomic traits, there was good concordance of effect size and direction (Fig. 2; *R*^2^ = 0.57).

**Fig. 3:**
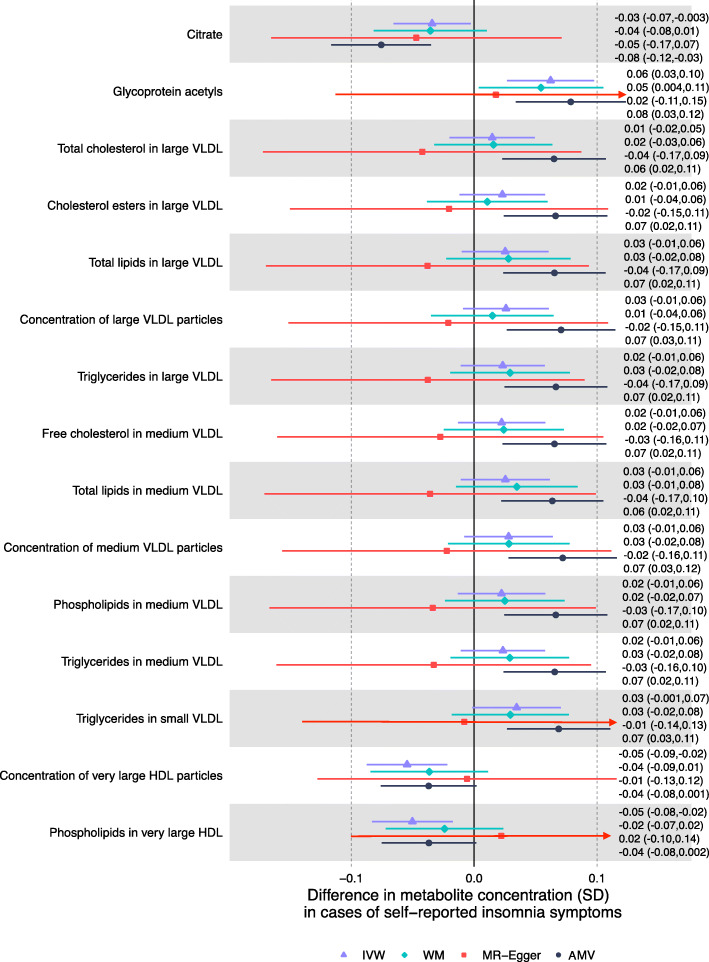
Mendelian randomization and age-, sex-, and BMI-adjusted multivariable regression analyses results for select associations of insomnia symptoms with NMR-derived metabolomic measures. Figure shows inverse variance weighted (IVW) Mendelian randomization, Mendelian randomization sensitivity (weighted median (WM) and MR-Egger), and adjusted multivariable (AMV) regression analysis results. Results presented were selected on the basis of passing multiple testing threshold for either IVW or AMV (*p* values < 0.0029). The estimates are the difference in mean metabolite (in standard deviation units) between those reporting usually versus sometimes/rarely/never experiencing insomnia symptoms. Abbreviations: AMV, adjusted (age, sex, BMI) multivariable regression; BMI, body mass index; IVW MR, inverse variance weighted Mendelian randomization; NMR, nuclear magnetic resonance; SD, standard error; VLDL, very low-density lipoprotein; WM, weighted median

Associations of insomnia symptoms with the 113 metabolomic traits passed the multiple testing threshold (*P* < 0.0029) for 13 in the AMV analyses, and for 3 in the MR analyses (glycoprotein acetyls passed the threshold in both). Based on our pre-specified definition of consistency (i.e. same direction and *p* value < 0.05 in MR for any AMV results reaching the corrected *p* value, and vice versa), we found consistent evidence from AMV and MR analyses for 4 associations. Specifically, usual vs. sometimes/rare/never insomnia symptoms lowered citrate (− 0.08SD [95% CI − 0.12, − 0.03] in AMV and − 0.03SD [− 0.07, − 0.003] in MR), increased glycoprotein acetyls (0.08SD [95% CI 0.03, 0.12] in AMV and 0.06 [0.03, 0.10] in MR), and lowered total very large HDL particles (− 0.04SD [− 0.08, 0.00] in AMV and − 0.05SD [− 0.09, − 0.02] in MR) and phospholipids in very large HDL particles (− 0.04SD [− 0.08, 0.002] in AMV and − 0.05SD [− 0.08, − 0.02] in MR) (Fig. 3). MR sensitivity analyses were generally consistent with the main IVW analyses though point estimates for the MR-Egger result with glycoprotein acetyls appeared weaker and that for phospholipids in very large HDL was weakly in the opposite direction. That said, as expected, confidence intervals were wide for all of the MR-Egger results (Fig. 3).

### Sleep duration

**Fig. 4:**
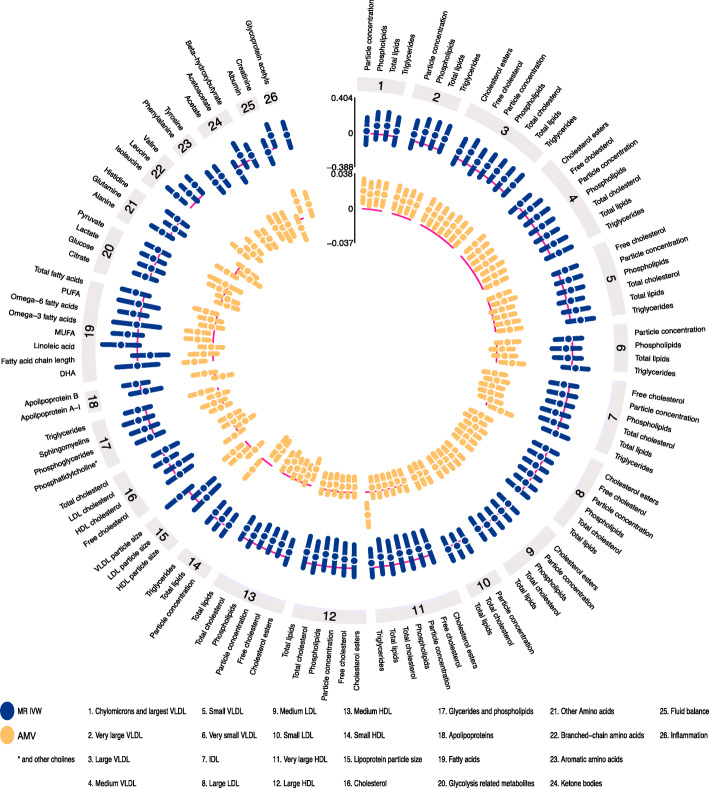
IVW Mendelian randomization estimates and age-, sex-, and BMI-adjusted multivariable regression estimates for the associations between total sleep duration and 113 NMR-derived metabolomic measures. Results are expressed as the difference in mean metabolite concentrations (in standard deviation units) for each 1 h greater reported total sleep duration. For visualization purposes, the axes have unequal scaling. Abbreviations: AMV, adjusted (age, sex, BMI) multivariable regression; BMI, body mass index; IDL, intermediate density lipoprotein; IVW MR, inverse variance weighted Mendelian randomization; LDL, low-density lipoprotein; NMR, nuclear magnetic resonance; VLDL, very large density lipoprotein

**Fig. 5:**
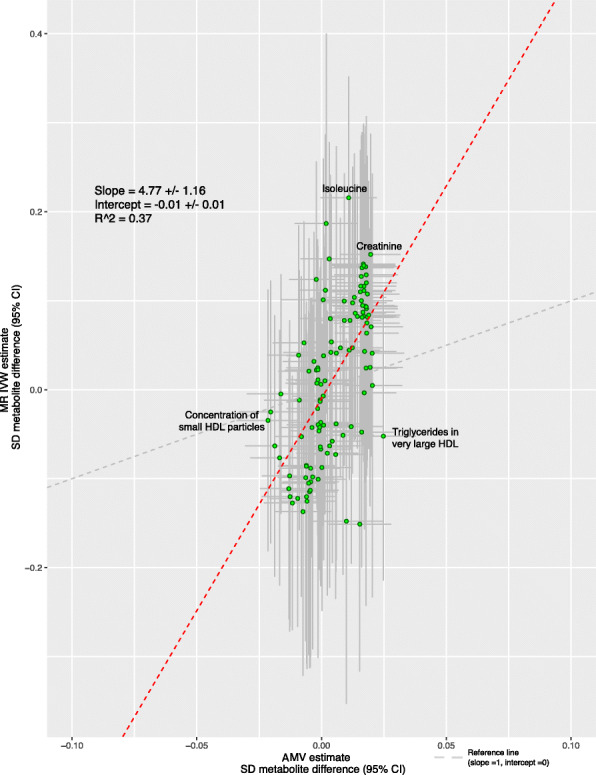
Comparison of the point estimates of the IVW Mendelian randomization and age-, sex-, and BMI-adjusted multivariable regression analyses for the associations between total sleep duration and 113 NMR-derived metabolomic measures. Each green dot in the scatter plot represents a metabolic trait and the positions of the dots are determined by the differences in mean metabolite concentrations (in standard deviation units) for each 1-h greater reported total sleep duration. These are estimated by Inverse variance weighted (IVW) Mendelian randomization (vertical axes) and age, sex, and BMI adjusted multivariable regression (horizontal axes). The vertical grey lines for each dot indicate the 95% confidence intervals (CI) for the Mendelian randomization estimates and the horizontal grey lines for each dot indicate the 95% CI for the adjusted multivariable regression estimates. A linear fit (red dashed line) summarizes the similarity between the two estimates. A slope of 1 with an intercept of 0 (dashed grey line), with all green dots sitting on that line (*R*^2^ = 1), would indicate identical magnitude and direction between the two methods. *R*^2^ indicates goodness of linear fit and is a measure of the consistency between the two estimates. Abbreviations: AMV, adjusted (age, sex, BMI) multivariable regression; BMI, body mass index; CI, confidence interval; IVW MR, inverse variance weighted Mendelian randomization, SD, standard deviation

Several associations of total sleep duration with metabolomics traits were directionally consistent in the AMV and MR analyses (Fig. 4). Where association directions were consistent, the MR results often had a stronger magnitude of association than the AMV results. Consistency of magnitude (as well as direction) was poor to moderate between the two methods (Fig. 5, *R*^2^ = 0.37).

**Fig. 6:**
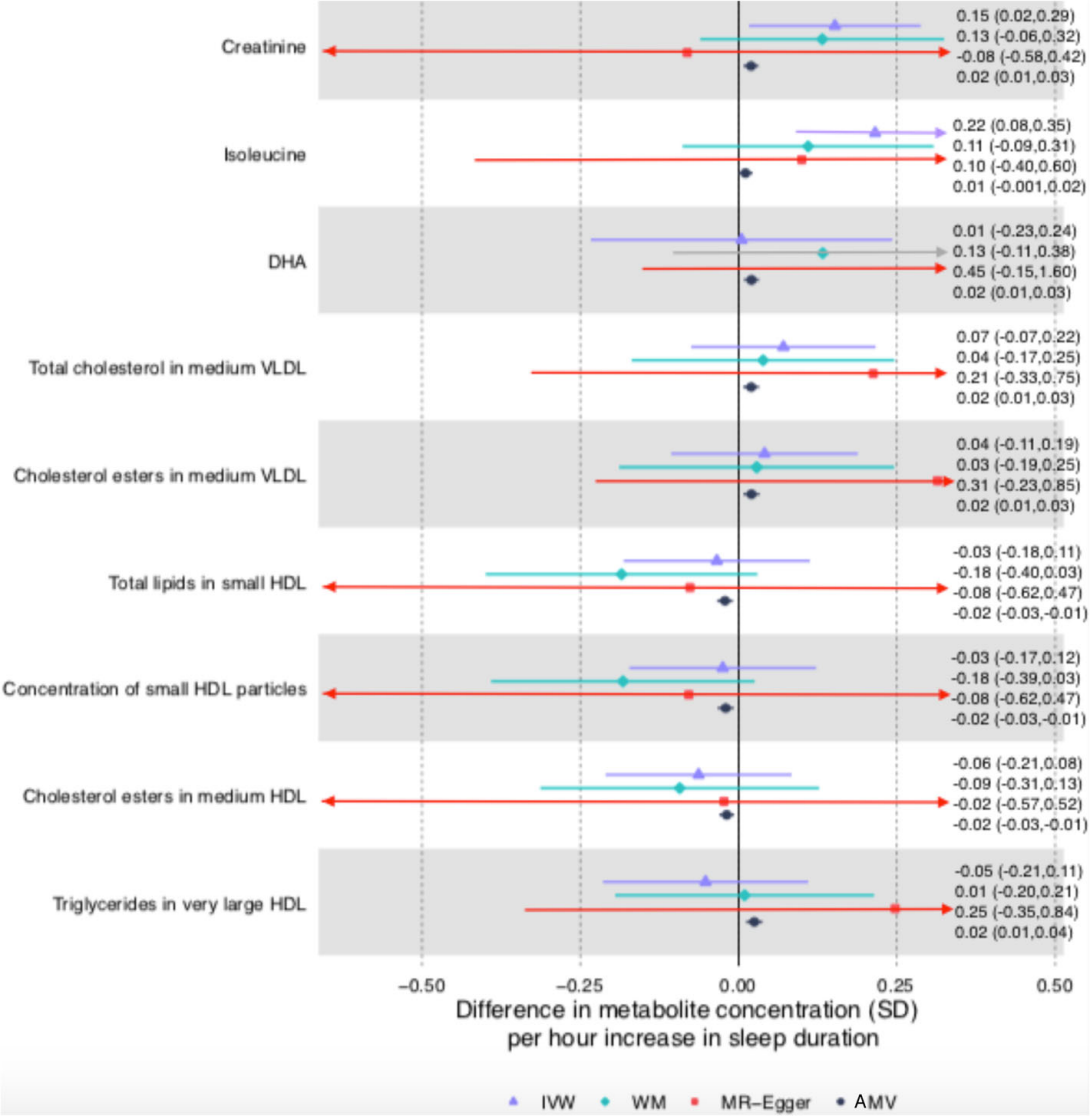
Mendelian randomization and age-, sex-, and BMI-adjusted multivariable regression analyses results for selected associations of total sleep duration with NMR-derived metabolomic measures. Figure shows inverse variance weighted (IVW) Mendelian randomization, Mendelian randomization sensitivity (weighted median (WM) and MR-Egger), and adjusted multivariable (AMV) regression analysis results. Results presented were selected on the basis of passing multiple testing threshold for either IVW or AMV (*p* values < 0.0029). The estimates are the difference in mean metabolite (in standard deviation units) per 1 h greater total sleep duration. Abbreviations: AMV, adjusted (age, sex, BMI) multivariable regression; BMI, body mass index; DHA, 22:6, docosahexaenoic acid; HDL, high-density lipoprotein; IVW MR, inverse variance weighted Mendelian randomization; NMR, nuclear magnetic resonance; SD, standard error; WM, weighted median

Associations for total sleep duration passed the multiple testing threshold for 8 of the 113 metabolomic trait associations in AMV analyses and one in MR. Only one of the 8 AMV associations replicated in the MR analyses (difference in mean creatinine for a 1 h longer sleep was 0.02SD [0.01, 0.03] in AMV and 0.15 [0.02, 0.29] in MR). Isoleucine was the one metabolite to pass the multiple testing threshold in IVW MR analyses, but it did not replicate in AMV analyses (0.01SD [− 0.001, 0.02] in AMV and 0.22 [0.08, 0.36] in MR analyses]) (Fig. 6). For the associations with creatinine, the weighted median MR result was consistent with that of the main (IVW) results but MR-Egger was in the opposite direction (though with very wide confidence intervals). For isoleucine, both MR sensitivity analyses had point estimates that were directionally, and in magnitude, similar to the main IVW MR results (Fig. 6).

In exploratory analyses, most associations of short sleep duration (< 7 h) were close to the null in both AMV and MR analyses, with very little overall agreement between the two methods (*R*^2^ = 0.09, Additional file 2: Figures S1 and S2). Two associations of short sleep passed the multiple testing corrected *p* value in AMV analyses (22:6 docosahexaenoic acid (DHA) and omega-3 fatty acids), with short sleep duration associated with lower levels for both of these; none passed the multiple testing threshold in the MR analyses. For docosahexaenoic acid (DHA) and omega-3 fatty acids, there was an inverse association in IVW MR analyses that had a larger effect estimate than in the AMV analyses but with wide confidence intervals that included the null (Additional file 2: Figure S3).

A total of 31 of the 113 metabolites passed the multiple testing threshold in the AMV analyses of long sleep duration (≥ 9 h), including higher concentrations of most extremely large, large and medium VLDL, triglycerides, and concentrations of glycoprotein acetyls and isoleucine (Additional file 2: Figures S4). MR analyses did not support a causal effect for any of these, with MR analysis point estimate close to the null or in the opposite direction (Additional file 2: Figure S6). We did not identify any metabolic traits passing the multiple testing threshold in IVW MR.

### Chronotype

**Fig. 7:**
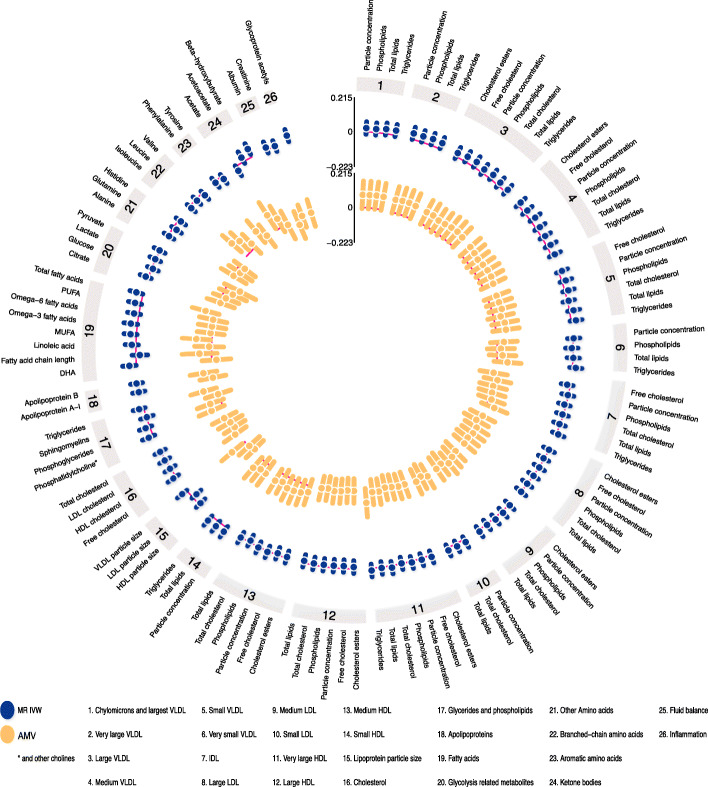
IVW Mendelian randomization estimates and age-, sex-, and BMI-adjusted multivariable regression estimates for the associations between chronotype and 113 NMR-derived metabolomic measures. Results are the difference in mean metabolite concentrations (in standard deviation units) between those reporting an evening versus morning preference. Abbreviations: AMV, adjusted (age, sex, BMI) multivariable regression; BMI, body mass index; IDL, intermediate density lipoprotein; IVW MR, inverse variance weighted Mendelian randomization; LDL, low-density lipoprotein; NMR, nuclear magnetic resonance; VLDL, very large density lipoprotein

**Fig. 8:**
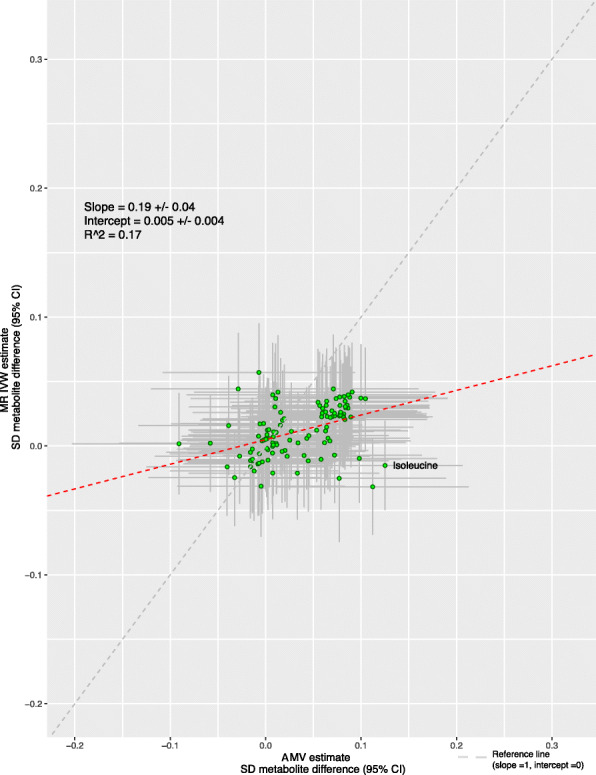
Comparison of the point estimates of the IVW Mendelian randomization and age-, sex-, and BMI-adjusted multivariable regression analyses for the associations between chronotype and 113 NMR-derived metabolomic measures. Each green dot in the scatter plot represents a metabolic trait and the positions of the dots are determined by the differences in mean metabolite concentrations (in standard deviation units) comparing those reporting an evening preference versus morning preference. These are estimated by Inverse variance weighted (IVW) Mendelian randomization (vertical axes) and age, sex, and BMI adjusted multivariable regression (horizontal axes). The vertical grey lines for each dot indicate the 95% confidence intervals (CI) for the Mendelian randomization estimates and the horizontal grey lines for each dot indicate the 95% CI for the adjusted multivariable regression estimates. A linear fit (red dashed line) summarizes the similarity between the two estimates. A slope of 1 with an intercept of 0 (dashed grey line), with all green dots sitting on that line (*R*^2^ = 1), would indicate identical magnitude and direction between the two methods. *R*^2^ indicates goodness of linear fit and is a measure of the consistency between the two estimates. Abbreviations: AMV, adjusted (age, sex, BMI) multivariable regression; BMI, body mass index; CI, confidence interval; IVW MR, inverse variance weighted Mendelian randomization, SD, standard deviation

**Fig. 9:**
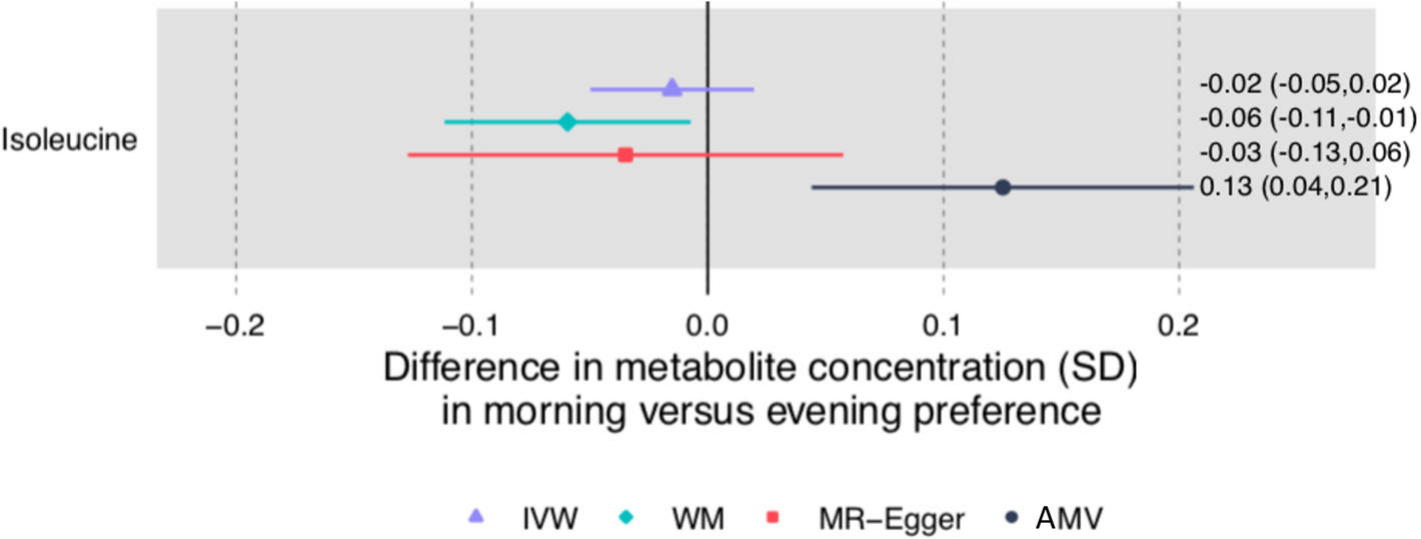
Mendelian randomization and age-, sex-, and BMI-adjusted multivariable regression analyses results for selected associations of chronotype with NMR-derived metabolomic measures. Figure shows inverse variance weighted (IVW) Mendelian randomization, Mendelian randomization sensitivity (weighted median (WM) and MR-Egger), and adjusted multivariable (AMV) regression analysis results. Results presented were selected on the basis of passing multiple testing threshold for either IVW or AMV (*p* values < 0.0029). The estimates are the difference in mean metabolite (in standard deviation units) comparing report of having an evening versus morning preference. Abbreviations: AMV, adjusted (age, sex, BMI) multivariable regression; BMI, body mass index; IVW MR, inverse variance weighted Mendelian randomization; NMR, nuclear magnetic resonance; SD, standard deviation; WM, weighted median

There was very little consistency in direction and magnitude of association between AMV and MR analyses of chronotype with the metabolomic traits (Figs. 7 and 8, *R*^2^ = 0.17). Chronotype was associated with isoleucine after multiple testing correction in the AMV analyses (difference in mean comparing evening to morning preference (0.13SD [0.04, 0.21]), but this was not supported in MR analyses (− 0.02 [− 0.05, 0.02])) (Fig. 9). No associations of chronotype with the metabolomics traits passed the multiple testing threshold in the IVW MR analyses.

### Between SNP-heterogeneity analyses

Across all four independent datasets, with MR results for the 5 sleep exposures and 113 metabolites, there were some Q-statistic *p* values that were less than the conventional threshold of 0.05. For most of these, their low *p* values were not seen for results across all four of the GWAS summary datasets. For results where we found MR evidence of an effect (i.e. for insomnia with citrate, glycoprotein acetyls, very large HDL particles, and phospholipids in very large HDL, and for sleep duration with creatinine and isoleucine) with two exceptions, there was no statistical evidence of between SNP heterogeneity in results for any of the independent datasets. For the effect of insomnia on glycoprotein acetyls, there was some evidence of between SNP heterogeneity in the MAGNETIC summary dataset (Q-statistic *p* value = 0.01; Supplementary Table S8) but not in other datasets. Notably, the MR-Egger intercept did not suggest evidence of any unbalanced pleiotropy (*p* = 0.47; Supplementary Table S8). For the effect of total mean sleep duration on isoleucine, there was some evidence of between SNP heterogeneity in one of the smaller GWAS (NEO; Q-statistic *p* = 2.3× 10^−5^; Supplementary Table S10), but this was not seen for results from other datasets and the MR-Egger intercept was very close to zero (*p* = 0.64; Supplementary Table S10).

## Discussion

With the present multi-cohort effort, we intended to identify the potential biochemical mechanisms linking sleep to cardiometabolic disease risk. We found consistent evidence with both AMV and MR that usually (vs. sometimes, rarely or never) experiencing insomnia symptoms cause lower concentrations of citrate, total very large HDL particles and phospholipids in very large HDL particles and higher concentrations of glycoprotein acetyls. There was little consistency between AMV and MR results for total habitual sleep duration across all metabolomic traits, though a longer total sleep duration was associated with higher concentrations of creatinine in both methods. For chronotype, whilst having an evening preference was associated with higher isoleucine concentrations at our multiple-testing threshold in the AMV analyses, MR analyses did not support causality. Chronotype did not pass multiple testing with any other metabolites. Therefore, our findings do not support the notion that sleep traits have widespread effects on the investigated metabolomic traits. Nevertheless, they suggest that insomnia symptoms may influence cardiometabolic disease (as previously shown in MR [17]) through increased inflammation and also result in lower citrate levels.

The lack of a more widespread impact of sleep traits on multiple metabolomic traits is in contrast with some experimental sleep studies, although direct comparisons are not possible. For example, targeted and untargeted mass spectrometry measurements performed in frequently sampled blood (every 2 h) from 12 healthy men revealed that 109 out of 171 metabolites exhibited a circadian rhythm [51]. Furthermore, in controlled experimental conditions, this circadian variation was maintained for 78 out of these 109 metabolites over a 24-h period of total sleep deprivation. For 27 metabolites, including some lipids (13 glycerophospholipids and 3 sphingolipids), as well as tryptophan, serotonin, taurine, and 8 acylcarnitines, marked acute increases in concentrations were observed during 24 h of sleep deprivation compared with the 24 h of habitual sleep [51]. Importantly, the MR analyses assessed long-term (lifelong), rather than acute, effects of a predisposition for unfavourable quality or quantity of sleep on metabolic disturbances, which could explain the generally stronger effects in the total sleep duration MR analyses.

Glycoprotein acetyls, which we identified as a novel trait potentially influenced by insomnia symptoms, are elevated in response to infection and inflammation. C-reactive protein (CRP) is the most widely recognized marker of acute and chronic inflammation in epidemiological studies. Whilst observational studies have shown that higher circulating CRP is associated with increased cardiovascular disease risk, MR studies suggest this is not a causal relationship [52, 53]. Glycoprotein acetyls have emerged as a potentially better measure of cumulative inflammation than CRP, since glycoprotein acetyls increase late in the inflammatory process and levels are relatively stable within individuals over many years [54, 55]. In AMV analyses in prospective cohorts, glycoprotein acetyls were positively associated with cardiovascular diseases and type 2 diabetes, independently of established risk factors and CRP [55]. If these associations are shown to be causal, then it is possible that cumulative chronic inflammation, as measured by glycoprotein acetyls, mediates the effect of insomnia on coronary heart disease identified in MR analyses [17]. However, we acknowledge that our results for the effect of insomnia on glycoprotein acetyls require replication in independent and larger studies and testing in ancestries other than Europeans.

The inverse association of insomnia symptoms with citrate in both AMV and MR analyses is novel. A recent narrative review highlighted the physiological control of plasma citrate concentrations in health and disease [56]*.* One possible mechanism through which insomnia might influence citrate is via the relationship of insomnia with night-time eating (which is also accompanied with higher night physical activity) [57], which would result in higher TCA cycle activity and consequently lower plasma citrate concentrations. However, despite a plausible role, there is a paucity of clinical and epidemiological studies of the effect of citrate levels on disease outcomes [56]. Citrate is converted to Acetyl-CoA by the enzyme ATP citrate lyase (ACLY). This action is on the path to cholesterol biosynthesis up stream of HMGCR, the enzyme that is the target of statins [58]. Both MR and RCT evidence show ACLY inhibition reduce LDLc levels and proportionately coronary heart disease risk by a similar amount to statins [59–61]. However, this provides only indirect evidence for a role of citrate on cardiovascular risk and it is notable that we found no strong evidence in this study of an effect of insomnia on LDLc. Therefore, the meaning of a possible effect of insomnia on citrate levels, and whether it mediates any effect of insomnia on cardiovascular disease risk is hard to discern. Whether our findings for citrate replicate would also be important to clarify.

We found evidence for associations of experiencing insomnia symptoms with higher concentrations of very large total HDL particles and phospholipids in very large HDL particles. MR and randomized controlled trials suggest that circulating HDL cholesterol is not causally related to cardiovascular disease [62–64]. The amount of cholesterol carried in HDL particles increases with increasing particle size and emerging evidence highlights the importance of considering size, structure, and composition of lipoprotein particles when exploring their effects on cardiovascular disease [65]. In AMV analyses, inverse associations of very large, large, medium, and small HDL particles with cardiovascular disease have been observed, but these attenuated to the null with adjustment for lipids used by clinicians [42]. Thus, the relevance of possible insomnia effects on very large HDL particle concentrations, and specifically phospholipids in these particles, is unclear and require additional studies.

We found evidence in both AMV and MR analyses of a possible association of longer total sleep duration with higher creatine concentrations, a biomarker used to estimate kidney function. Established cardiovascular risk factors, such as high blood pressure and type 2 diabetes, are associated with higher creatinine concentrations [66]. Findings from multivariable regression suggest that the association of kidney function with cardiovascular disease largely reflects confounding and/or reverse causality [67]. Thus, our observations possibly suggest that longer sleep duration is an additional risk factor for chronic kidney disease rather than cardiovascular diseases, though we acknowledge MR sensitivity analyses did not support a causal effect. It is also possible longer sleep duration results in higher creatine concentrations via dehydration, though we might then have expected similar effects on more of the other metabolite concentrations. We also found a novel association of longer total sleep duration with the branched-chain amino acid isoleucine in MR analyses, though this association was not observed in the AMV analyses. This raises the possibility of masking (negative) confounding in the AMV analyses, though it would be surprising for this to specifically affect this one branch chain amino acid. It is also possible that the MR analyses are biased by unbalanced pleiotropy, although the MR-Egger intercept being very close to zero would argue against that. Higher concentrations of branched-chain amino acids, including isoleucine, are associated with increased risk of cardiovascular disease [42], though this has not been explored in MR studies. MR analyses supports a causal effect of the branched-chain amino acids on type 2 diabetes [68], and our results suggest that longer total sleep duration may mediate some of this effect. Although the mechanism of action how sleep induces higher isoleucine concentrations, speculatively, this might be the result of protein degradation required for gluconeogenesis. More research is required to further elaborate on this hypothesis.

Key strengths of our study are its novelty and the comparison of results from the largest AMV study of sleep traits with multiple circulating metabolomic measures [22] with equivalent results from MR. We harmonized questionnaire-based sleep data across all contributing studies and the NMR metabolomic platform was consistent across studies in both the AMV and MR analyses. We were able to increase the power of our two-sample MR analyses by combining unpublished summary-level GWAS results from three cohorts (total *N* = 13,693) with those of the largest published GWAS of the same NMR platform (*N* = 24,925) to date [35]. Two-sample MR assumes that the two samples are from the same underlying population and independent of each other. Given all GWAS were undertaken in adults of European ancestry and the lack of overlap in studies contributing to the metabolite GWAS with any of the sleep trait GWAS, we are confident this assumption is largely met. Most observed differences in mean metabolomic concentrations were close to the null, and in general (true) null results are less subject to bias than non-null results [69].

Important limitations include the lack of statistical power, particularly to explore possible non-linear associations for sleep duration. The platform misses a high proportion of currently quantifiable metabolites in human serum/plasma, including markers of energy balance, microbiota metabolism, vitamins, co-factors, and xenobiotics, that may be influenced by sleep traits [51]. Still, the NMR platform used in the analyses covers considerably more of the lipidome than conventional clinical chemistry measures (total cholesterol, LDL-C, HDL-C, and triglycerides) that have previously been explored and in addition includes amino acids, glycolysis metabolites, ketone bodies, and an inflammatory marker. Whilst we adjusted for age, sex, and BMI, the results obtained in multivariable-adjusted regression may be exaggerated by residual confounding from unobserved confounders such as socioeconomic position, smoking, and physical activity. As the AMV results were cross-sectional, it is also possible that variation in metabolomic traits influences sleep patterns, and some of the multivariable regression results not verified in MR are due to reverse causality. In addition, we restricted the analyses to cohorts containing mostly European participants (one cohort contributing to AMV meta-analysis, HELIUS, included non-European participants, whereas all MR analyses were restricted to Europeans). This reduces the potential for population stratification to bias our MR analyses, but hampers generalization of our findings to other ancestry groups. In addition, the cohorts contributing in the AMV meta-analysis vary in participant characteristics, in particular by age. In the cohorts used in the AMV analyses only, 2.4% reported taking medication to aid sleep. This very small proportion means these are very unlikely to have introduced any bias into our analyses. However, it is known that many prescribed, and over the counter medications, can influence sleep, and in our study, as in others exploring sleep, we were not able to do a detailed assessment of all medications. The MR results which reflect a potential lifelong genetic tendency should be less influenced by medication use. Furthermore, the use of questionnaire-based data on sleep traits might have increased measurement error. As people do not know the concentrations of their circulating metabolites or genetic variants related to those, such error is likely to be random and would therefore be expected in both analyses to bias towards the null. Accelerometer-based sleep measures could be useful to further explore the effects we have studied, but previous observational and genetic studies suggest only moderate agreement between questionnaire-based and accelerometer-based sleep duration [21, 70], and it is unclear whether the two are measuring the same construct. The MR results may have been influenced by weak instrument bias, which, if present, would be expected to bias results towards the null. The very large F-statistics for our main analyses (2537 to 13,967), and even for our secondary analyses of short and long duration (208 and 646, respectively), suggest that weak instrument bias is unlikely to have a major impact. Sensitivity analyses exploring possible bias due to directional horizontal pleiotropy were mostly consistent with the main IVW findings, though MR-Egger estimates were imprecise as expected with this method which is statistically less efficient than the main IVW method.

## Conclusions

Taken together, our findings do not suggest widespread metabolic disruption caused by sleep traits. However, the evidence for possible effects of insomnia symptoms on glycoprotein acetyls and citrate and longer total sleep duration on creatinine and isoleucine might explain some of the effects, found in MR analyses, of these sleep traits on cardiometabolic diseases. These warrant further investigation.

## Supplementary Information

**Additional file 1: Table S1.** Characteristics of the study populations in the multivariable-adjusted regression analyses. **Table S2.** Genetic instruments for insomnia. **Table S3.** Genetic instruments for total sleep duration. **Table S4.** Genetic instruments for short sleep duration. **Table S5.** Genetic instruments for long sleep duration. **Table S6.** Genetic instruments for chronotype. **Table S7.** Multivariable-adjusted results for insomnia symptoms. **Table S8.** Mendelian Randomization results for insomnia symptoms. **Table S9.** Multivariable-adjusted results for total sleep duration. **Table S10.** Mendelian Randomization results for total sleep duration. **Table S11.** Multivariable-adjusted results for short sleep duration. **Table S12.** Mendelian Randomization results for short sleep duration. **Table S13.** Multivariable-adjusted results for long sleep duration. **Table S14.** Mendelian Randomization results for long sleep duration. **Table S15.** Multivariable-adjusted results for chronotype. **Table S16.** Mendelian Randomization results for chronotype.

**Additional file 2: Figure S1.** IVW Mendelian randomization estimates, and age, sex and BMI-adjusted multivariable regression estimates for the associations between short sleep duration and 113 NMR derived metabolites. **Figure S2.** Comparison of the point estimates of the IVW Mendelian randomization and age, sex and BMI-adjusted multivariable regression analyses for the associations between short sleep duration and 113 NMR derived metabolites. **Figure S3.** Mendelian randomization and age, sex and BMI-adjusted multivariable regression analyses results for select associations of short sleep duration with NMR metabolites. **Figure S4.** IVW Mendelian randomization estimates and age, sex and BMI adjusted multivariable regression estimates for the associations between long sleep duration and 113 NMR derived metabolites. **Figure S5.** Comparison of the point estimates of the IVW Mendelian randomization and age, sex and BMI-adjusted multivariable regression analyses for the associations between long sleep duration and 113 NMR derived metabolites. **Figure S6A.** Mendelian randomization and age, sex and BMI-adjusted multivariable regression analyses results for select associations of long sleep duration with NMR metabolites. **Figure S6B.** Mendelian randomization and age, sex and BMI adjusted multivariable regression analyses results for select associations of long sleep duration with NMR metabolites.

## Data Availability

The summary-level datasets used and/or analysed during the current study are available from the corresponding authors on reasonable request.

## Abbreviations

AGO: Active and Healthy Ageing
AMV: Adjusted multivariable
CI: Confidence interval
CRP: C-reactive protein
DHA: Docosahexaenoic acid
DWFS: Dutch Hunger Winter Families Study
GWAS: Genome-wide association study
HDL-C: High-density lipoprotein cholesterol
HELIUS: Healthy Life in an Urban Setting
IVW: Inverse-variance weighted
LDL-C: Low-density lipoprotein cholesterol
LUMINA: Leiden University Migraine Neuro-Analysis
MR: Mendelian randomization
NEO: Netherlands Epidemiology of Obesity
NMR: Nuclear magnetic resonance
NTR: Netherlands Twin Register
RS: Rotterdam Study
SD: Standard deviation
VLDL: Very large density lipoprotein
