# Supplementary material for: Investigating the relationships between unfavourable habitual sleep and metabolomic traits: evidence from multi-cohort multivariable regression and Mendelian randomization analyses
Source: BMC Med. 2021 Mar 18;19:69. doi: 10.1186/s12916-021-01939-0 (PMC7971964; doi:10.1186/s12916-021-01939-0)
Supplement: Supplementary file 2 — Additional file 2: Figure S1. IVW Mendelian randomization estimates, and age, sex and BMI-adjusted multivariable regression estimates for the associations between short sleep duration and 113 NMR derived metabolites. Figure S2. Comparison of the point estimates of the IVW Mendelian randomization and age, sex and BMI-adjusted multivariable regression analyses for the associations between short sleep duration and 113 NMR derived metabolites. Figure S3. Mendelian randomization and age, sex and BMI-adjusted multivariable regression analyses results for select associations of short sleep duration with NMR metabolites. Figure S4. IVW Mendelian randomization estimates and age, sex and BMI adjusted multivariable regression estimates for the associations between long sleep duration and 113 NMR derived metabolites. Figure S5. Comparison of the point estimates of the IVW Mendelian randomization and age, sex and BMI-adjusted multivariable regression analyses for the associations between long sleep duration and 113 NMR derived metabolites. Figure S6A. Mendelian randomization and age, sex and BMI-adjusted multivariable regression analyses results for select associations of long sleep duration with NMR metabolites. Figure S6B. Mendelian randomization and age, sex and BMI adjusted multivariable regression analyses results for select associations of long sleep duration with NMR metabolites. [file 12916_2021_1939_MOESM2_ESM.docx]

**ADDITIONAL FILE 2: SUPPLEMENTAL FIGURES**

**Investigating the relationships between unfavorable sleep and metabolomic traits: evidence from multi-cohort multivariable regression and Mendelian randomization analyses**

Maxime M Bos (1, 2)^*^, Neil J Goulding (3, 4)^*^, Matthew A Lee (3, 4), Amy Hofman (2), Mariska Bot (5), René Pool (6, 7), Lisanne S Vijfhuizen (8), Xiang Zhang (9, 10), Chihua Li (11), Rima Mustafa (12), Matt J Neville (13, 14), Ruifang Li-Gao (15), Stella Trompet (1), Marian Beekman (16), Nienke R Biermasz (17), Dorret I Boomsma (6, 7), Irene de Boer (18), Constantinos Christodoulides (14), Abbas Dehghan (12, 19, 20), Ko Willems van Dijk (8, 17, 21), Ian Ford (22), Mohsen Ghanbari (2), Bastiaan T Heijmans (16), M Arfan Ikram (2), J Wouter Jukema (23,24), Dennis O Mook-Kanamori (15, 25), Fredrik Karpe (13, 14), Annemarie I Luik (2), L.H. Lumey (11,16), Arn M.J.M. van den Maagdenberg (8, 18), Simon P Mooijaart (1), Renée de Mutsert (15), Brenda W.J.H. Penninx (5), Patrick CN Rensen (17, 21), Rebecca C Richmond (3, 4), Frits R Rosendaal (15), Naveed Sattar (26), Robert A Schoevers (27), P Eline Slagboom (16, 28), Gisela M Terwindt (18), Carisha S Thesing (5), Kaitlin H Wade (3, 4), Carolien A Wijsman (1), Gonneke Willemsen (6, 7), Aeilko H. Zwinderman (29), Diana van Heemst (1)^#^, Raymond Noordam (1)^#^, Deborah A Lawlor (3, 4, 30)^#^

^*^ Joint first authors

^#^ Joint senior authors

1. Department of Internal Medicine, Section of Gerontology and Geriatrics, Leiden University Medical Center, Leiden, the Netherlands.

2. Department of Epidemiology, Erasmus MC University Medical Center Rotterdam, Rotterdam, the Netherlands.

3. MRC Integrative Epidemiology Unit at the University of Bristol, Bristol, UK.

4. Population Health Sciences, Bristol Medical School, University of Bristol, Bristol, UK.

5. Amsterdam UMC, Vrije Universiteit, Psychiatry, Amsterdam Public Health research institute, Amsterdam, the Netherlands.

6. Amsterdam Public Health Research Institute, Amsterdam, the Netherlands.

7. Department of Biological Psychology, Vrije Universiteit Amsterdam, Amsterdam, the Netherlands.

8. Department of Human Genetics, Leiden University Medical Center, Leiden, the Netherlands.

9. Department of Experimental Vascular Medicine, Amsterdam UMC, University of Amsterdam, Amsterdam, The Netherlands.

10. Human and Animal Physiology, Wageningen University, Wageningen, The Netherlands.

11. Department of Epidemiology, Mailman School of Publc Health, Columbia University, New York, USA.

12. Department of Epidemiology and Biostatistics, School of Public Health, Imperial College London, London, UK.

13. NIHR Oxford Biomedical Research Centre, Oxford University Hospitals Foundation Trust, Oxford, UK.

14. Radcliffe Department of Medicine, Oxford Centre for Diabetes, Endocrinology, and Metabolism, University of Oxford, Oxford, UK.

15. Department of Clinical Epidemiology, Leiden University Medical Center, Leiden, the Netherlands.

16. Molecular Epidemiology, Department of Biomedical Data Sciences, Leiden University Medical Center, Leiden, The Netherlands.

17. Department of Internal Medicine, Division of Endocrinology, Leiden University Medical Center, Leiden, the Netherlands.

18. Department of Neurology, Leiden University Medical Center, Leiden, the Netherlands.

19. Dementia Research Institute at Imperial College London, London W2 1PG, UK.

20. MRC Centre for Environment and Health, School of Public Health, Imperial College, London, UK.

21. Einthoven Laboratory for Experimental Vascular Medicine, Leiden University Medical Center, Leiden, the Netherlands.

22. Robertson Center for Biostatistics, University of Glasgow, UK.

23. Department of Cardiology, Leiden University Medical Center, Leiden, the Netherlands.

24. Netherlands Heart Institute, Utrecht, the Netherlands

25. Department of Public Health and Primary Care, Leiden University Medical Center, Leiden, the Netherlands.

26. BHF Glasgow Cardiovascular Research Centre, Faculty of Medicine, Glasgow, UK.

27. University of Groningen, University Medical Center Groningen, Department of Psychiatry, Groningen, the Netherlands.

28. Max Planck Institute for Biology of Ageing, Cologne, Germany.

29. Department of Clinical Epidemiology, Biostatistics, and Bioinformatics, Amsterdam UMC, University of Amsterdam, The Netherlands.

30. NIHR Bristol Biomedical Research Centre, Bristol, UK.

**Supplementary Figures**

**Figure S1:** **IVW Mendelian randomization estimates, and age, sex and BMI-adjusted multivariable regression estimates for the associations between short sleep duration and 113 NMR derived metabolites**

*Results are the difference in mean metabolite concentrations (in standard deviation units) between those with compared to without short sleep duration. For visualization purposes the axes have unequal scaling*. *Abbreviations:* *AMV, adjusted (age, sex, BMI) multivariable regression; BMI, body mass index; IDL, intermediate density lipoprotein; IVW MR, Inverse variance weighted Mendelian randomization; LDL, low density lipoprotein; NMR, nuclear magnetic resonance; VLDL, very large density lipoprotein.*

**Figure S2:** **Comparison of the point estimates of the IVW Mendelian randomization and age, sex and BMI-adjusted multivariable regression analyses for the associations between short sleep duration and 113 NMR derived metabolites**

*Each green dot in the scatter plot represents a metabolic trait and the positions of the dots are determined by the differences in mean metabolite concentrations (in standard deviation units) between those with compared to without short sleep duration. These are estimated by inverse variance weighted (IVW) Mendelian randomization (vertical axes) and age, sex and BMI adjusted multivariable regression (horizontal axes). The vertical grey lines for each dot indicate the 95% confidence intervals (CI) for the Mendelian randomization estimates and the horizontal grey lines on each dot indicate the 95% CI for the adjusted multivariable regression estimates. A linear fit (red dashed line) summarizes the similarity between the two estimates. A slope of 1 with an intercept of 0 (dashed grey line), with all green dots sitting on that line (R^2^ = 1), would indicate identical magnitude and direction between the two methods. R^2^ indicates goodness of linear fit and is a measure of the consistency between the two estimates. Abbreviations: AMV, adjusted (age, sex, BMI) multivariable regression; BMI, body mass index; CI, confidence interval; DHA, 22:6, docosahexaenoic acid; IVW MR, Inverse variance weighted Mendelian randomization, SD, standard deviation.*

**Figure S3:** **Mendelian randomization and age, sex and BMI-adjusted multivariable regression analyses results for select associations of short sleep duration with NMR metabolites.**

*Figure shows inverse variance weighted (IVW) Mendelian randomization, Mendelian randomization sensitivity (weighted median (WM) and MR-Egger) and adjusted multivariable (AMV) regression analysis results. Results presented were selected on the basis of passing multiple testing threshold for either IVW or AMV (p-values < 0.0029).* *The estimates are the difference in mean metabolite (in standard deviation units) between those with and without short sleep duration.* Abbreviations: *AMV, adjusted (age, sex, BMI) multivariable regression; BMI, body mass index; DHA,* 22:6, docosahexaenoic acid; *IVW MR, Inverse variance weighted Mendelian randomization; NMR, nuclear magnetic resonance; SD, standard deviation; WM, Weighted Median.*

**Figure S4:** **IVW Mendelian randomization estimates and age, sex and BMI adjusted multivariable regression estimates for the associations between long sleep duration and 113 NMR derived metabolites**.

**** *Results are the difference in mean metabolite concentrations (in standard deviation units) between those with compared to without long sleep duration. Abbreviations: AMV, adjusted (age, sex, BMI) multivariable regression; BMI, body mass index; IDL, intermediate density lipoprotein; IVW MR, Inverse variance weighted Mendelian randomization; LDL, low density lipoprotein; NMR, nuclear magnetic resonance; VLDL, very large density lipoprotein.*

**Figure S5:** **Comparison of the point estimates of the IVW Mendelian randomization and age, sex and BMI-adjusted multivariable regression analyses for the associations between long sleep duration and 113 NMR derived metabolites**.

*Each green dot in the scatter plot represents a metabolic trait and the positions of the dots are determined by the differences in mean metabolite concentrations (in standard deviation units) between those with compared to without long sleep duration. These are estimated by inverse variance weighted (IVW) Mendelian randomization (vertical axes) and age, sex and BMI adjusted multivariable regression (horizontal axes). The vertical grey lines for each dot indicate the 95% confidence intervals (CI) for the Mendelian randomization estimates and the horizontal grey lines on each dot indicate the 95% CI for the adjusted multivariable regression estimates. A linear fit (red dashed line) summarizes the similarity between the two estimates. A slope of 1 with an intercept of 0 (dashed grey line), with all green dots sitting on that line (R^2^ = 1), would indicate identical magnitude and direction between the two methods. R^2^ indicates goodness of linear fit and is a measure of the consistency between the two estimates.* Abbreviations: *AMV, adjusted (age, sex, BMI) multivariable regression; BMI, body mass index; CI, confidence interval; IVW MR, Inverse variance weighted Mendelian randomization; NMR, nuclear magnetic resonance; SD, standard error.*

**Figure S6A:** **Mendelian randomization and age, sex and BMI-adjusted multivariable regression analyses results for select associations of long sleep duration with NMR metabolites.**

*Figure shows inverse variance weighted (IVW) Mendelian randomization, Mendelian randomization sensitivity (weighted median (WM) and MR-Egger) and adjusted multivariable (AMV) regression analysis results. Results presented were selected on the basis of passing multiple testing threshold for either IVW or AMV (p-values < 0.0029).* *The estimates are the difference in mean metabolite (in standard deviation units) between those with and without long sleep duration.* ** and chylomicrons.* Abbreviations: *AMV, adjusted (age, sex, BMI) multivariable regression; BMI, body mass index; IVW MR, Inverse variance weighted Mendelian randomization; NMR, nuclear magnetic resonance; SD, Standard Error; VLDL, very low density lipoprotein; WM, Weighted Median.*

**Figure S6B:** **Mendelian randomization and age, sex and BMI adjusted multivariable regression analyses results for select associations of long sleep duration with NMR metabolites.**

*Figure shows inverse variance weighted (IVW) Mendelian randomization, Mendelian randomization sensitivity (weighted median (WM) and MR-Egger) and adjusted multivariable (AMV) regression analysis results. Results presented were selected on the basis of passing multiple testing threshold for either IVW or AMV (p-values < 0.0029).* *The estimates are the difference in mean metabolite (in standard deviation units) between those with and without long sleep duration.* ** and chylomicrons. Abbreviations:* Abbreviations: *AMV, adjusted (age, sex, BMI) multivariable regression; BMI, body mass index;* acid; CI, confidence interval; *IVW MR, Inverse variance weighted Mendelian randomization; MUFA, mono-unsaturated fatty acids; NMR, nuclear magnetic resonance; SD, Standard Error; VLDL, very low density lipoprotein; WM, Weighted Median.*
